# Screening differentially expressed genes between endometriosis and ovarian cancer to find new biomarkers for endometriosis

**DOI:** 10.1080/07853890.2021.1966087

**Published:** 2021-08-19

**Authors:** Zhenzhen Lu, Ying Gao

**Affiliations:** Department of Gynaecology and Obstetrics, Union Hospital, Tongji Medical College, Huazhong University of Science and Technology, Wuhan, China

**Keywords:** Differentially expressed genes, endometriosis, ovarian cancer, biomarkers

## Abstract

**Aim:**

Endometriosis is one of the most common reproductive system diseases, but the mechanisms of disease progression are still unclear. Due to its high recurrence rate, searching for potential therapeutic biomarkers involved in the pathogenesis of endometriosis is an urgent issue.

**Methods:**

Due to the similarities between endometriosis and ovarian cancer, four endometriosis datasets and one ovarian cancer dataset were downloaded from Gene Expression Omnibus (GEO) database. Differentially expressed genes (DEGs) were identified, followed by gene ontology (GO), Kyoto Encyclopedia of Genes and Genomes (KEGG) pathway and protein–protein interaction (PPI) analyses. Then, we validated gene expression and performed survival analysis with ovarian serous cystadenocarcinoma (OV) datasets in TCGA/GTEx database, and searched for potential drugs in the Drug-Gene Interaction Database. Finally, we explored the miRNAs of key genes to find biomarkers associated with the recurrence of endometriosis.

**Results:**

In total, 104 DEGs were identified in the endometriosis datasets, and the main enriched GO functions included cell adhesion, extracellular exosome and actin binding. Fifty DEGs were identified between endometriosis and ovarian cancer datasets including 11 consistently regulated genes, and nine DEGs with significant expression in TCGA/GTEx. Only *IGHM* had both significant expression and an association with survival, three module DEGs and two significantly expressed DEGs had drug associations, and 10 DEGs had druggability.

**Conclusions:**

*ITGA7*, *ITGBL1* and *SORBS1* may help us understand the invasive nature of endometriosis, and *IGHM* might be related to recurrence; moreover, these genes all may be potential therapeutic targets.

**KEY MESSAGE**
- This manuscript used a bioinformatics approach to find target genes for the treatment of endometriosis.
- This manuscript used a new approach to find target genes by drawing on common characteristics between ovarian cancer and endometriosis.
- We screened relevant therapeutic agents for target genes in the drug database, and performed histological validation of target genes with both expression and survival analysis difference in cancer databases.

## Introduction

Endometriosis is defined by endometrial tissue located outside of the uterine cavity [1,2]. Approximately, 6–10% of women of reproductive age are affected by this disease, and infertility and pelvic pain are the primary symptoms [3]. Dysmenorrhoea, irregular uterine bleeding and dyspareunia also occur frequently in those patients. Endometriosis mainly occurs in the ovary, followed by the ligaments of the pelvic, the fallopian tract, the umbilicus, the abdominal wall, the cervical-vaginal area, the urinary tract, and the eyes, lung and brain. This characteristic of distant metastasis is similar to that of tumours, but the pathogenesis has yet to be fully elucidated. Influencing factors are extensive and include environmental, genetic, stem cell, immunogenicity, lymphatic and vascular dissemination factors [4,5]. Gynaecologic surgery is the main treatment, while other treatments include nonsteroidal anti-inflammatory drugs, progestins, combined oral contraceptives and GnRH-a injection [6]. Regardless of these treatments, endometriosis has a high recurrence rate.

Ovarian cancer is one of the three major malignant tumours in obstetrics and gynaecology, and the diagnosis and treatment of ovarian cancer are relatively mature and prevalent, in particular, nanomedicines offer new prospects for ovarian cancer treatment [7]. Endometriosis and ovarian cancer have certain similarities in terms of invasion, angiogenesis and adhesion, but the difference is that endometriosis does not have the infinite proliferation observed in ovarian cancer. Several studies have shown that endometriosis is one of the risk factors for ovarian cancer [8], and a proportion of ovarian cancers have been shown to originate from 0.5 to 1% of cases of ovarian endometriosis [9,10]. Ovarian endometriosis may present a risk for ovarian malignant lesions according to gene expression and miRNA alterations [11,12], and is always managed with the prevention of carcinogenesis [13]. Immunity and inflammation are thought to be strongly associated with carcinogenicity [14,15]; however, no studies have shown how long ovarian cancer takes to develop from ovarian endometriosis. All evidence shows relationships between endometriosis and ovarian cancer; thus, screening differentially expressed genes (DEGs) between ovarian cancer and endometriosis may provide an alternative route to identify the mechanisms involved in the carcinogenesis and recurrence of endometriosis.

In recent years, microarrays have been widely used to identify therapeutic targets and candidate biomarkers by investigating the alteration of gene expression at a genome-wide level [16,17]. With the integration of bioinformatics technology and clinical treatment [18–21], a number of studies have been published, including studies on endometriosis. DEGs such as *NR4A1* [22], *ITPR1* [23], *CXCL12* [24], *HSPA5*, *ENO2* and *TJP1* [25] have been proven important in the progression of endometriosis. miRNAs, such as miR-200b-3p [26], miR-1266-5p, and miR-200a-3p [27], and even circular RNAs (circRNAs), for example, has-circ-0003380, has-circ-0020093 and has-circ-0008016, were all significantly overexpressed in endometriosis [28]. In our study, we drew on the common features of two different diseases to identify key DEGs, which may provide a new direction for treatment.

## Materials and methods

### Data collection

The Gene Expression Omnibus (GEO, http://www.ncbi.nlm.nih.gov/geo/) is a freely available international public repository for next-generation sequencing-based functional genomic datasets and high-throughput microarrays. It also provides users with several web-based tools to query, analyse and visualize data [29], such as GEO2R. Four endometriosis datasets, GSE5108, GSE7305, GSE11691 and GSE25628, and one ovarian cancer dataset, GSE14407 were obtained from GEO. The GSE5108 dataset contained 11 ectopic endometrium samples and 11 eutopic endometrium samples. GSE7305 contained 10 ectopic endometrium samples and 10 normal endometrium samples. GSE11691 contained nine ectopic endometrium samples and nine normal endometrium samples. GSE25628 contained eight ectopic endometrium samples and eight normal endometrium samples. GSE14407 contained 12 normal samples and 12 tumour samples.

### Identification of DEGs

GEO2R (http://www.ncbi.nlm.nih.gov/geo/geo2r/) [29] is an R-based website that helps users perform GEO data analysis, and identify genes that are differentially expressed [30,31]. The four endometriosis datasets described above were analysed using GEO2R, and GSE14407 was analysed by RStudio (version 4.0.4). The limma package was applied to identify the DEGs between cancer and normal groups, with the GPL570 [HG-U133_Plus_2] Affymetrix Human Genome U133 Plus 2.0 Array. The statistically significant settings were | log (fold change) | >1 and *p* value <.05.

### Gene ontology (GO), signalling pathway and protein–protein interaction (PPI) networks

GO (http://geneontology.org) is the most widely used knowledge base and provides structured knowledge regarding the functions of genes and gene products [32], including biological processes (BPs), cellular components (CCs) and molecular functions (MFs) [33]. GO and Kyoto Encyclopedia of Genes and Genomes (KEGG) pathway analyses were performed using the web-based DAVID tool (version 6.8, http://www.david.niaid.nih.gov), which is for the functional annotation of DEGs [34]. In addition, we also used R to perform GO analysis of 104 DEGs, and to ensure the reliability of our results. Next, PPI networks were predicted using by STRING (version 11.0, https://string-db.org/), which was applied to explore the physical and functional associations between the DEGs [35], with a combined score >0.4 (medium confidence). PPIs were visualized using Cytoscape software (version 3.8.1) [36], and the Molecular Complex Detection plugin (MCODE, version 2.0.0) was used to find the most significant modules, with the following settings: degree cut-off = 2, node score cut-off = 0.2, max depth = 100 and *k*-score = 2.

### Validation of DEGs between endometriosis and ovarian cancer on GEPIA in TCGA/GTEx databases

To further select for precise biomarkers, we performed gene expression level and survival analysis with Gene Expression Profiling Interactive Analysis (GEPIA, http://gepia.cancer-pku.cn/), a web-based tool to deliver fast and customizable functionalities based on Cancer Genome Atlas (TCGA) and Genotype-Tissue Expression (GTEx) data, and provided key interactive and customizable functions [37]. Gene expression validation involved 514 samples of ovarian serous cystadenocarcinoma (OV) datasets built in TCGA/GTEx database (tumour: 426 normal: 88), with thresholds |log2FC| ≥1 and *p* value <.01, setting jitter size =0.4. Overall survival (OS) and disease-free survival (DFS) were assessed in OV datasets, and time data were sorted into low-expression and high-expression groups by the median transcripts per kilobase (TPM).

### Possible drugs for target genes

The Drug-Gene Interaction Database (DGIdb, http://www.dgidb.org) is a web resource that helps users interpret the results of genome-wide studies in the context of the druggable genome [38]. DGIdb organizes genes of the druggable genome into known drug interactions and potentially druggable targets [38]. We input module DEGs of endometriosis and significantly evaluated DEGs in DGIdb to find potentially druggable DEGs.

### Immunofluorescence

Ectopic endometrium, eutopic endometrium and normal endometrium were fixed, embedded and sliced. After deparaffinizing and rehydrating the paraffin sections [39,40], they were placed in a repair box filled with citric acid antigen retrieval buffer (pH 6.0) for antigen retrieval. Next, sections were placed in 3% hydrogen peroxide and incubated at room temperature for 25 min to block endogenous peroxidase activity, followed by serum blocking with 3% BSA (Servicebio G5001, Wuhan, China) for 30 min at room temperature. Anti-human IgM rabbit monoclonal antibody (1:1000 dilution; HUABIO, Cambridge, MA) was incubated overnight at 4 °C, followed by an incubation with secondary antibody at room temperature for 50 min. After the addition of secondary antibody, the sections were incubated with DAPI (Servicebio G1012, Wuhan, China) solution for 10 min at room temperature, and then spontaneous fluorescence quenching reagent was added and incubated for 5 min. Then, cover slips were mounted with anti-fade mounting medium, and images were captured by fluorescence microscopy.

## Results

### DEG identification

**Figure 1.**
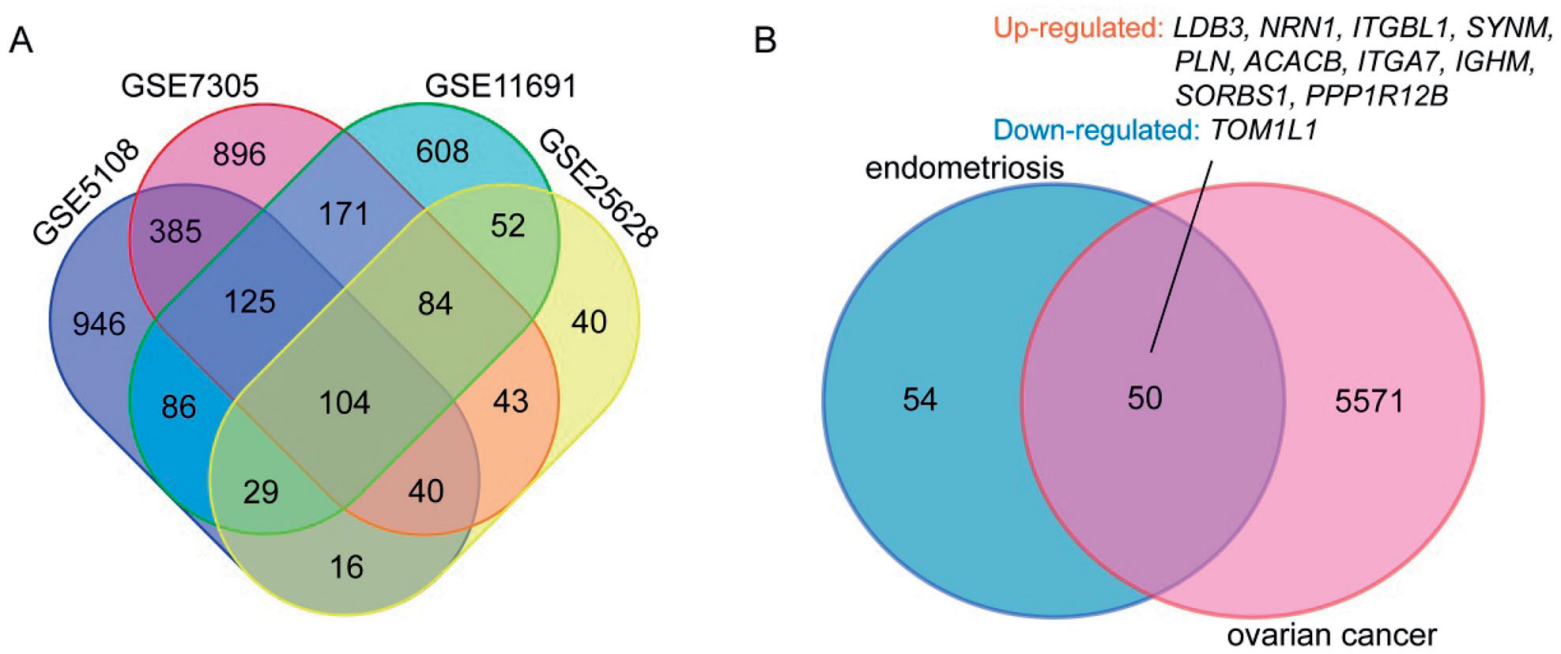
Venn diagram. (A) DEGs of endometriosis were selected with a fold change >1 and *p* value <.05 among the expression profiling sets GSE5108, GSE7305, GSE11691 and GSE25628. The four datasets showed an overlap of 104 genes. (B) DEGs of endometriosis and ovarian cancer datasets showed an overlap of 50 genes including 10 up-regulated and one down-regulated DEGs.

After standardization, DEGs associated with endometriosis (1846 in GSE5108, 2633 in GSE7305, 1513 in GSE11691 and 509 in GSE25628) were identified, as were DEGs associated with ovarian cancer (6887 in GSE14407). There were 104 genes among the four endometriosis datasets as shown in the Venn diagram (Figure 1(A)), including 84 consistently upregulated DEGs and 19 consistently downregulated DEGs. The DEGs behaved differently due to the heterogeneity of humans (Table 1). The overlap contained 50 DEGs, and only 11 DEGs had consistent regulation, including 10 upregulated DEGs and one downregulated DEG (Figure 1(B)).

**Table 1.** Identified DEGs in four endometriosis datasets, but *PLA2G5* had the different regulatory.

Category	Gene symbol
Upregulated	*KCNMA1, LDB3, WISP2, FRY, GHR, GATA6, MEIS2, TRIL, MGP, GPM6A, RERGL, NRN1, ITGBL1, MYLK, CSGALNACT1, LYVE1, CPA3, LHFP, PDE10A, ADIRF, SGCD, FILIP1L, RNASE1, CCDC69, MYL9, ACACB, DMD, ATRNL1, CPE, ITGA7, PCOLCE2, SYNM, LY96, HSD17B6, PDLIM5, NGF, HPR, FAM129A, PLN, PLA2G2A, DCLK1, CFH, RCAN2, IGHM, FZD7, TMEM47, CHL1, SLIT3, MYH11, IRAK3, AGTR1, TNS1, FMO1, C7, PDE1A, RGS2, RGS5, EPHA3, PPP1R12B, PDE2A, NFASC, CLDN5, PLXDC2, ACKR1, SORBS1, CLU, COL14A1, AEBP1, ITM2A, LRRN3, AQP1, CCL21, FMO2, ADH1B, ARHGAP6, FABP4, FRZB, PDLIM3, PPP1R3C, LTC4S, ACTA2, SYNPO, GEM, PTGIS*
Downregulated	*PTPN3, SORD, KLRC2/KLRC1, BUB1, ALDH3B2, ACSL5, PLS1, PRR5-ARHGAP8, GRHL2, GINS3, MPZL2, TOM1L1, HMGCR, KIAA1324, PPM1H, CWH43, SLC15A2, FOXA2, MAP7*
Inconsistency	*PLA2G5*

### GO and KEGG enrichment analyses of DEGs in endometriosis

**Figure 2.**
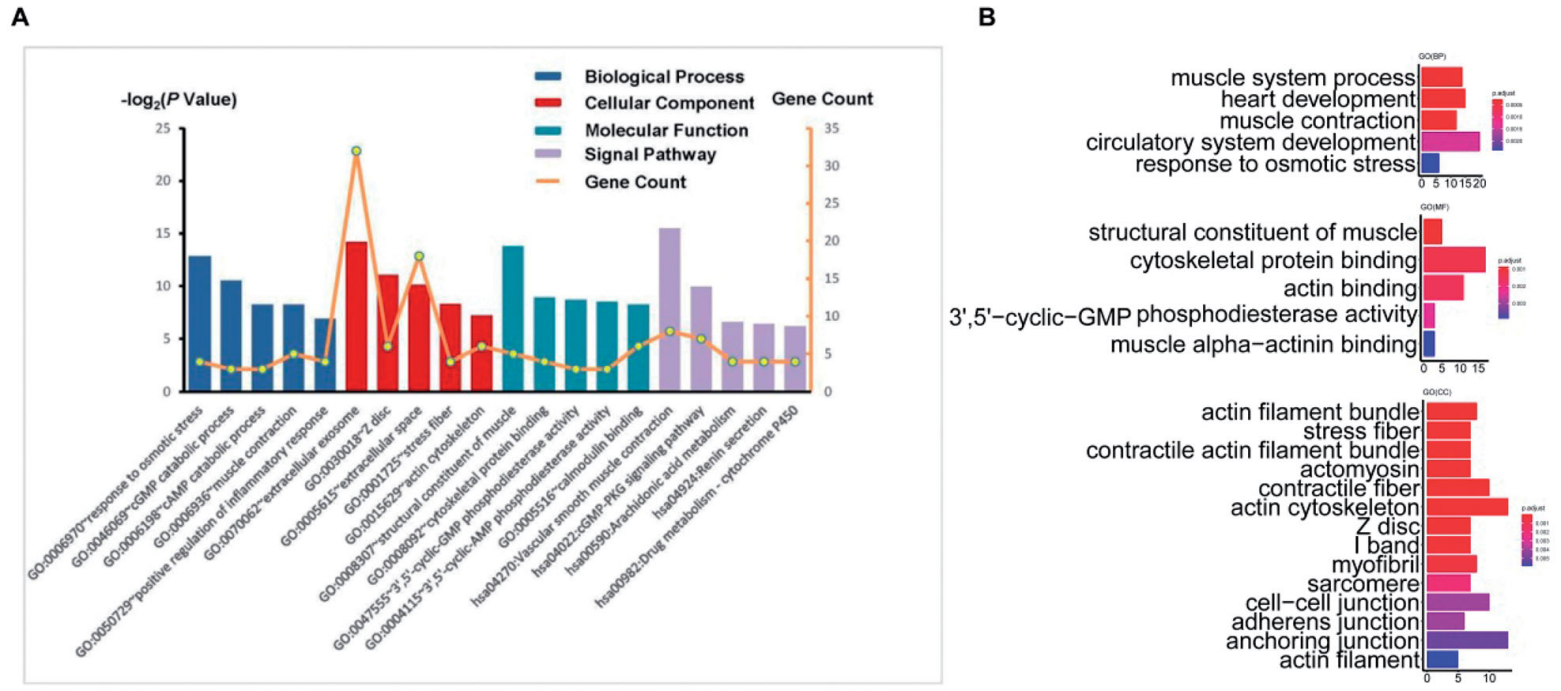
Most significant GO analysis of 104 DEGs by using DAVID (A) and R (B).

We identified the top five significant GO and signalling pathways with the criterion of a *p* value <.05 (Figure 2(A)). Then, we analysed the most enriched GO functions (Table 2). Among the upregulated, BP was mostly enriched in cell adhesion, muscle contraction and positive regulation of inflammatory response; CC was mainly enriched in extracellular exosome, plasma membrane and extracellular space; and MF was significantly enriched in actin binding, calmodulin binding and structural constituent of muscle. KEGG pathway analysis revealed that DEGs were mainly enriched in vascular smooth muscle contraction and the cGMP-PKG signalling pathway. The downregulated DEGs were mainly involved in response to osmotic stress and metabolic pathways. GO function analysis was also performed by R, and more results for BP, MF and CC were obtained, but we only showed the top functions in the diagram (Figure 2(B)). The main functions were roughly the same for the two methods, but we could not obtain KEGG results in the R analysis, as the gene number was too small. Thus, it seemed that DAVID was more advantageous, but the key DEGs involved in cell adhesion in the two methods were consistent.

**Table 2.** Top three most enriched BP, CC, MF and signal pathway analysis of 104 DEGs.

Terms	Category	Description	Count	*p* Value	Genes
*Upregulated*					
GO:0007155	BP	Cell adhesion	7	.020985707	*CHL1, ITGBL1, ITGA7, SORBS1, LYVE1, EPHA3, WISP2*
GO:0006936	BP	Muscle contraction	5	.001622453	*ACTA2, MYH11, SORBS1, MYL9, MYLK*
GO:0050729	BP	Positive regulation of inflammatory response	4	.004920582	*FABP4, PDE2A, PLA2G2A, AGTR1*
GO:0070062	CC	Extracellular exosome	28	6.09E–05	*GPM6A, IGHM, CFH, PCOLCE2, COL14A1, FAM129A, ADIRF, AEBP1, HPR, CLU, WISP2, MYLK, AQP1, C7, CHL1, MYH11, PLA2G2A, PLXDC2, LYVE1, RNASE1, ACTA2, CLDN5, NFASC, FABP4, KCNMA1, MGP, CPE, ITM2A*
GO:0005886	CC	Plasma membrane	28	.020719801	*GPM6A, IGHM, NRN1, FAM129A, LY96, AQP1, GHR, TMEM47, RGS5, RGS2, SGCD, CHL1, DMD, FZD7, PLA2G2A, PDE2A, SORBS1, LYVE1, CLDN5, NFASC, KCNMA1, AGTR1, CPE, ITGA7, ACKR1, PPP1R12B, ITM2A, EPHA3*
GO:0005615	CC	Extracellular space	17	2.87E–04	*CPA3, IGHM, NRN1, PTGIS, CFH, CCL21, COL14A1, PLA2G2A, LY96, AEBP1, CLU, WISP2, GHR, ACTA2, FRZB, CPE, SLIT3*
GO:0003779	MF	Actin binding	7	.00137143	*KCNMA1, DMD, SORBS1, SYNPO, PDLIM5, TNS1, MYLK*
GO:0005516	MF	Calmodulin binding	6	.001438965	*RGS2, PDE1A, MYH11, AEBP1, GEM, MYLK*
GO:0008307	MF	Structural constituent of muscle	5	3.36E–05	*SYNM, PDLIM3, MYH11, DMD, MYL9*
hsa04270	KEGG	Vascular smooth muscle contraction	7	1.01E–04	*ACTA2, KCNMA1, PLA2G2A, AGTR1, PPP1R12B, MYL9, MYLK*
hsa04022	KEGG	cGMP-PKG signalling pathway	7	5.18E–04	*RGS2, PLN, PDE2A, KCNMA1, AGTR1, MYL9, MYLK*
hsa04921	KEGG	Oxytocin signalling pathway	4	.074230056	*RGS2, PPP1R12B, MYL9, MYLK*
*Downregulated*					
GO:0006970	BP	Response to osmotic stress	2	.018076066	*SORD, MAP7*
hsa01100	KEGG	Metabolic pathways	4	.041843478	*ALDH3B2, SORD, ACSL5, HMGCR*

### PPI networks and the most significant modules of endometriosis

**Figure 3.**
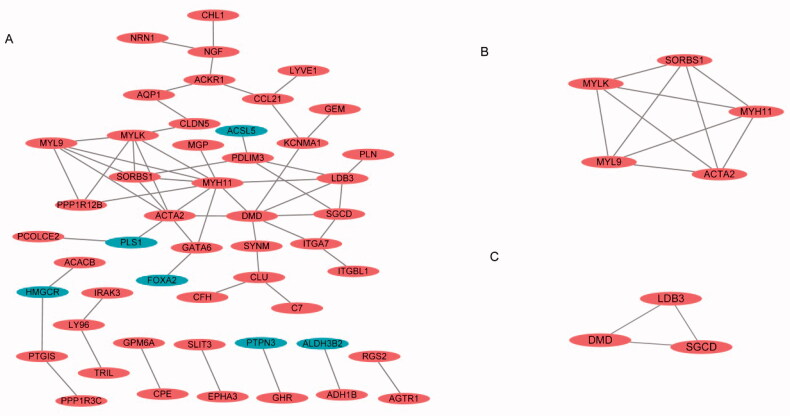
PPI network and the most significant module of DEGs of four endometriosis datasets. (A) The PPI network of DEGs was constructed using Cytoscape. (B, C) The most significant module was obtained by MCODE.

A total of 104 DEGs were uploaded into the STRING website (https://string-db.org/cgi/) and analysed by Cytoscape, with a setting score >0.4 (medium confidence), with options such as hiding disconnected nodes and showing input protein names selected for the construction (Figure 3(A)). The two most significant modules were clustered via MCODE. Module 1 was made up of five upregulated DEGs, and module 2 consisted of three upregulated DEGs (Figure 3(B,C)).

### Validation of the 11 DEGs in TCGA/GTEx

**Figure 4.**
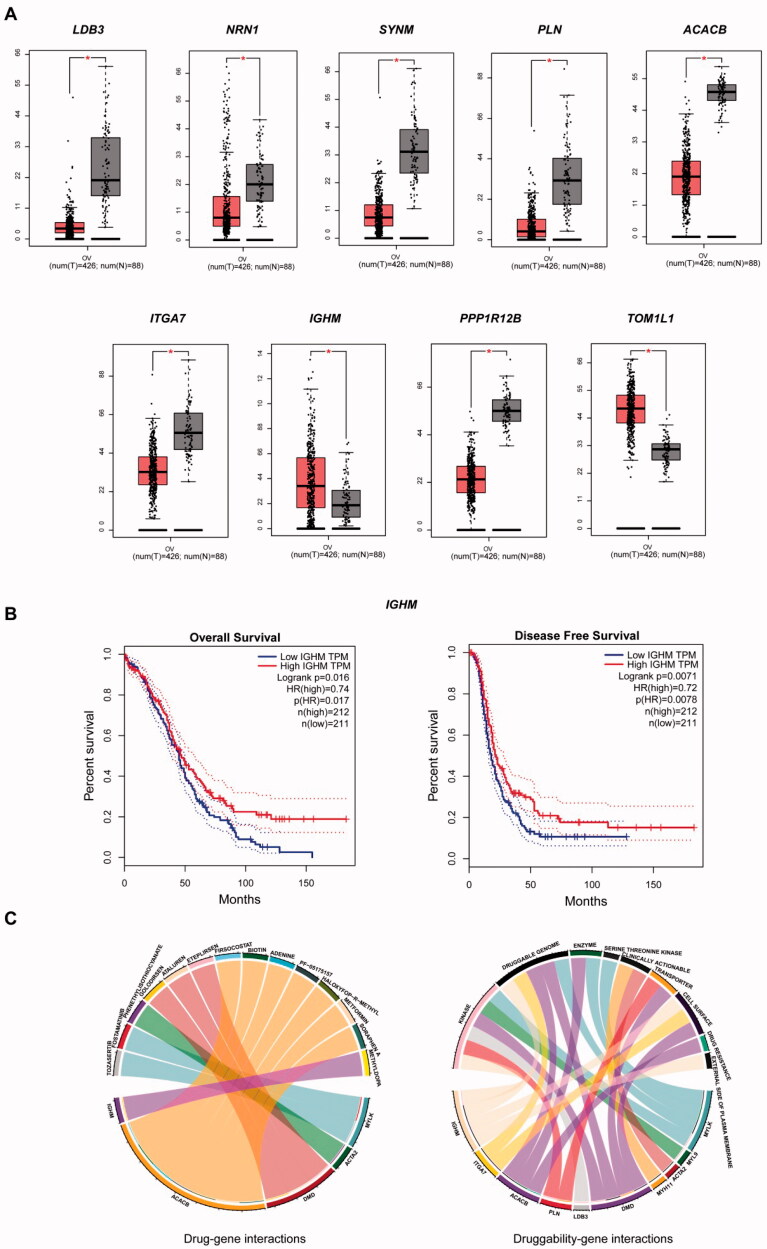
(A) Significant expression of nine DEGs with OV built in TCGA/GTEx datasets. (B) Significant OS and DFS analysis of *IGHM* between high expression and low expression on GEPIA. (C) Drug–gene interactions of three module DEGs and two significant expressed DEGs in DGIdb database; druggability of 10 DEGs and their nine kinds of drug categories.

The 11 DEGs were significantly enriched in protein binding and cell adhesion, and the signalling pathways were mainly enriched in dilated cardiomyopathy and the insulin signalling pathway (Table 3). For validation of the OV build in TCGA/GTEx, we found that only nine DEGs had significant expression in OV (Figure 4(A)). In addition, only *IGHM* had a significant difference between high expression and low expression in OS and DFS (Figure 4(B)). This candidate gene was significantly enriched in the regulation of extracellular exosomes and extracellular space (Table 2).

**Table 3.** Most significantly GO and signalling pathway of 11 DEGs.

Terms	Category	Description	*p* Value	Gene symbols
GO:0005515	MF	Protein binding	.005371	*TOM1L1, IGHM, SYNM, PLN, ITGA7, LDB3, SORBS1, PPP1R12B, ACACB*
GO:0008092	MF	Cytoskeletal protein binding	.022527	*LDB3, SORBS1*
GO:0007155	BP	Cell adhesion	.029014	*ITGBL1, ITGA7, SORBS1*
GO:0043086	BP	Negative regulation of catalytic activity	.043789	*PLN, ACACB*
GO:0007160	BP	Cell-matrix adhesion	.052336	*ITGA7, SORBS1*
GO:0007229	BP	Integrin-mediated signalling pathway	.057432	*ITGBL1, ITGA7*
GO:0030018	CC	Z disc	.06291	*LDB3, PPP1R12B*
hsa05414	KEGG	Dilated cardiomyopathy	.047967	*PLN, ITGA7*
hsa04910	KEGG	Insulin signalling pathway	.077878	*SORBS1, ACACB*

### Possible drugs for target genes

We input eight module DEGs and nine significantly expressed DEGs involved in the DGIdb database to identify drug–gene interactions and potential druggable gene targets. *MYLK*, *ACTA2* and *DMD* were associated with six kinds of drugs for endometriosis, and four of which had been validated by researchers, *ACACB* and *IGHM* were associated with eight kinds of drugs, three of which had been approved by researchers (Table 4). Ten of drugs were present in nine drug categories (Figure 4(C)).

**Table 4.** Drugs corresponding to DEGs in DGIdb database.

Gene	Drug	Approved	Score	Types	Sources	PMIDs
*MYLK*	TOZASERTIB	–	0.81	Inhibitor	DTC	19035792
	FOSTAMATINIB	Yes	0.19	Inhibitor	DrugBank	26516587
*ACTA2*	PHENETHYLISOTHIOCYANATE	–	2.14	–	DrugBank	21838287
*DMD*	GOLODIRSEN	–	132.55	Inducer	DrugBank|PharmGKB|FDA	29301272|31576784|30171533| 24554202
	ATALUREN	Yes	6.31	–	TTD	–
	ETEPLIRSEN	Yes	37.87	–	PharmGKB|FDA	–
*ACACB*	FIRSOCOSTAT	–	4.06	Allosteric modulator	GuideToPharmacology	–
	BIOTIN	Yes	2.71	Cofactor	DrugBank	16772434|17477831
	ADENINE	–	8.12	–	DrugBank	2880560|10592235|17139284| 8318018|17016423| 12829626
	PF-05175157	–	8.12	–	TTD	–
	HALOXYFOP-R-METHYL	–	16.23	–	DrugBank	10592235
	METFORMIN	Yes	0.2	–	TTD	–
	SORAPHEN A	–	32.46	–	DrugBank	10592235|17139284|17016423
*IGHM*	METHYLDOPA	Yes	0.72	–	DrugBank	23896426

### Immunofluorescence

**Figure 5.**
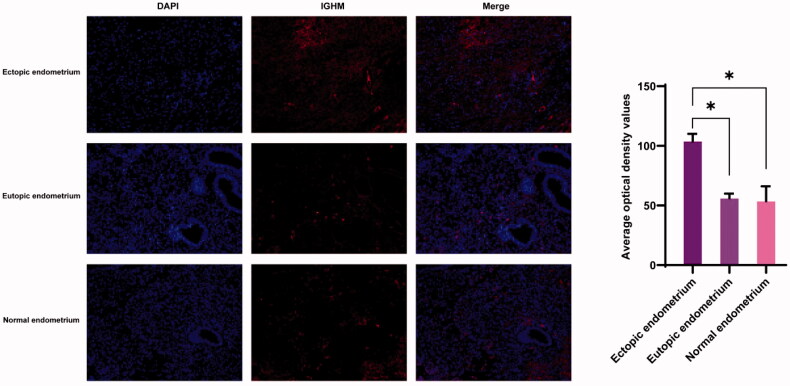
Immunofluorescence of ectopic endometrium, eutopic endometrium and normal endometrium.

Three sets of human tissue were collected for verification. In Figure 5, the expression of *IGHM* in endometrium, eutopic endometrium and normal endometrium was labelled by red fluorescence. The expression of *IGHM* was significantly higher in ectopic endometrium than in eutopic and normal endometrium, while there was no significant difference in its expression between eutopic and normal endometrium.

## Discussion

Endometriosis is an oestrogen-dependent disease associated with pelvic pain and reduced fertility [41,42], and has a complex aetiology, influenced by both genetic and environmental factors [43]. Relationships between endometriosis and ovarian cancer have been established, such as inflammatory response, vascular proliferation, distant invasion and associated increased levels of serum CA125. Endometriosis is a risk factor for ovarian cancer [44] and can transform to an atypical form and even to malignancy in 0.7–2.5% of cases [45]. In this study, the similarity in distant progression between endometriosis and ovarian cancer was used to find targets for the treatment of endometriosis.

The datasets used in our study have been widely used in other studies, suggesting that the results analysed with these datasets are supported by credibility. Research involving GSE5108 [46] is the most original sequencing analysis in this dataset, which only lists the genes with large variations in fold, and it indicated that cell adhesion associated genes may contribute to the adhesive and invasive properties of ectopic endometrium, consistent with our study. The GSE11691 [24,25], GSE7305 [24], GSE25628 [25] and GSE14407 had all been submitted to GO, KEGG and PPI analyses. These studies all selected the functions of DEGs ranked at the top by |log2FC|. Of course, this selection helped to obtain certain key functions, whereas in our study, we did not focus only on the magnitude with |log2FC|. As expression was not limited to |log2FC|, we focussed on the common DEGs of the two diseases with similar properties, and then selected the DEGs associated with adhesion function, to more precisely screen the target DEGs for our corresponding studies.

By taking the intersection of the four endometriosis datasets, we obtained 104 DEGs, and we obtained two clusters on endometriosis through the construction of PPI networks. *SORBS1*, *MYLK*, *MYH11*, *MYL9* and *ACAT2* were involved in cluster 1, and *LDB3*, *DMD* and *SGCD* were involved in cluster 2 (Figure 3). Eight DEGs may play important roles in the development of endometriosis, and three of the eight DEGs were associated with drugs in the drug database DGIdb (Table 4, Figure 4(C)). To identify target DEGs that may be involved in the recurrence of endometriosis, 11 DEGs with consistent up- and downregulation were identified among the 50 DEGs shared by endometriosis and ovarian cancer, and validations were performed in TCGA/GTEx. Nine DEGs had significant expressions; *LDB3, NRN1, SYNM, PLN, ACACB, ITGA7, IGHM, PPP1R12B* and *TOM1L1*, all of which were involved in the function of protein binding, and only *ACACB* and *IGHM* were identified as druggable targets in DGIdb (Table 4, Figure 4(C)). These druggable targets are pending future cellular and animal studies.

**Figure 6.**
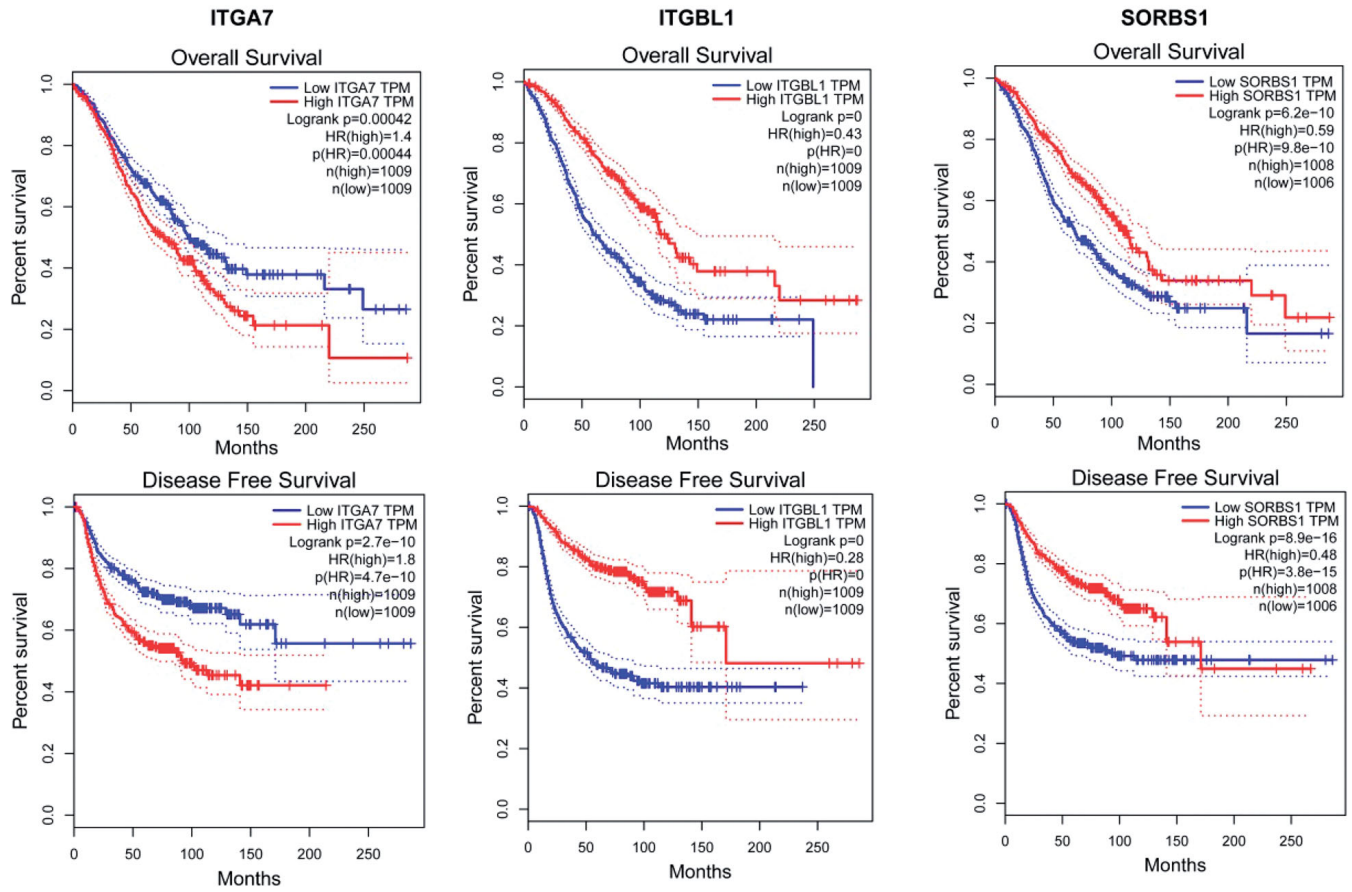
Significant OS and DFS analysis of *ITGA7*, *ITGBL1* and *SORBS1* between high expression and low expression among breast invasive carcinoma (BRCA), cervical squamous cell carcinoma and endocervical adenocarcinoma (CESC), ovarian serous cystadenocarcinoma (OV), uterine corpus endometrial carcinoma (UCEC) and uterine carcinosarcoma (UCS) on GEPIA.

**Figure 7.**
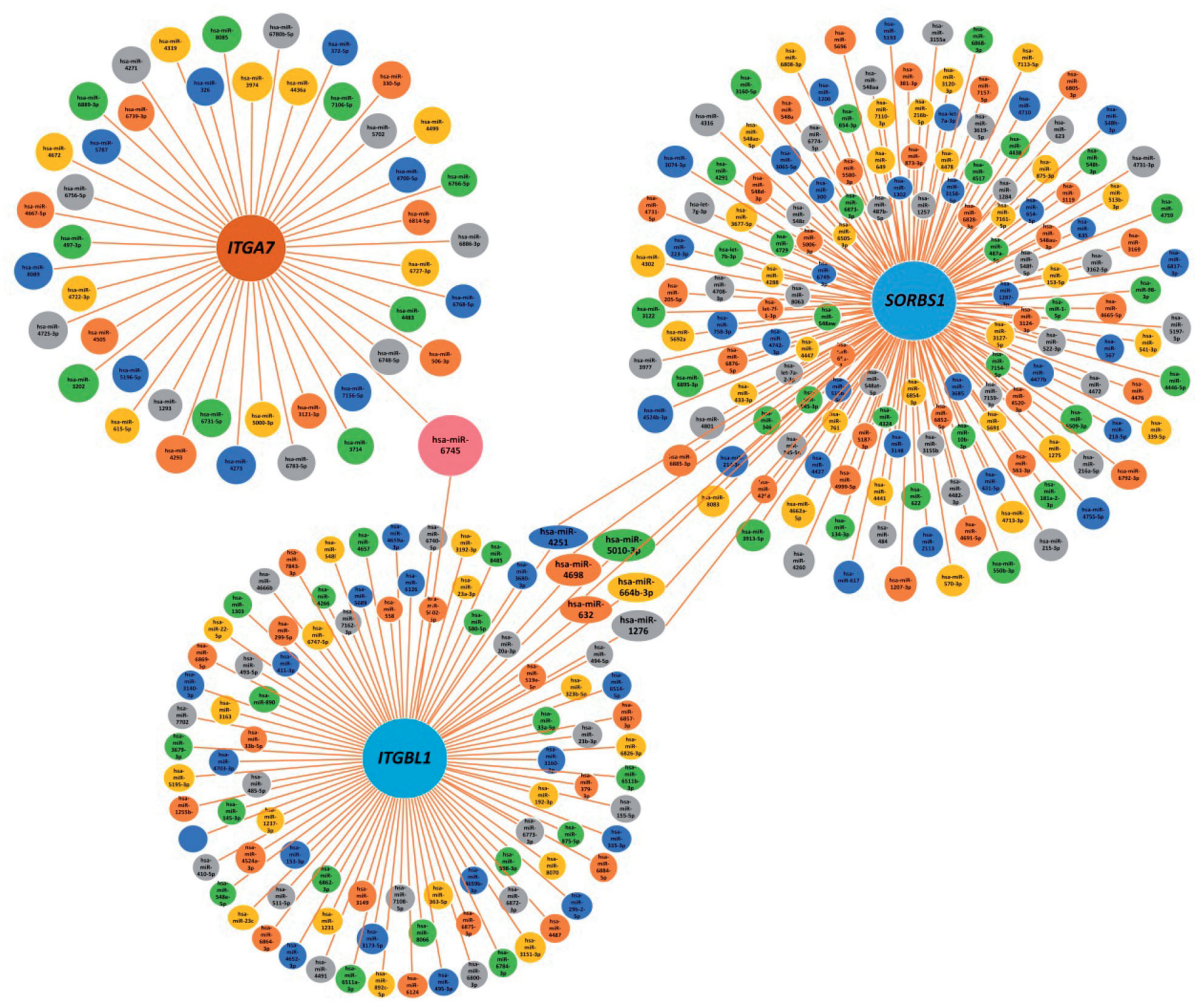
miRNAs and overlap of *ITGA7*, *ITGBL1* and *SORBS1* in miRDB, TargetScan and miWalk databases.

By analysing the GO functions of these 11 DEGs, we found that *ITGA7*, *ITGBL1* and *SORBS1* were mainly involved in cell adhesion (Table 3). We observed that *ITGA7* had a direct interaction with *ITGBL1* in PPI network (Figure 3), which suggested that they might be coexpressed, and all three genes were upregulated DEGs that regulated cell proliferation, invasion and migration in cancers [47–51]. It is of great significance to analyse their survival in obstetrics- and gynaecology-related tumours (Figure 6). *ITGA7* regulates cell proliferation via the PTK2-PI3K-Akt signalling pathway and is negatively associated with clinical outcomes in hepatocellular carcinoma [52], and via the laminin-integrin α7β1 signalling pathway in mechanical ventilation-induced pulmonary fibrosis [53]. Upregulation of *ITGBL1* predicted poor prognosis and promoted chemoresistance in ovarian cancer [54] and activated fibroblasts using extracellular vesicles (EVs) via NF-κB signalling. Moreover, it promoted epithelial-to-mesenchymal transition (EMT) of colorectal cancer (CRC) cells [55] and had the same characteristics in ovarian cancer [51] and prostate cancer [50]. *SORBS1* overexpression promoted CRC growth and migration via inhibition of *AHNAK* expression [56], while *SORBS1* was downregulated in breast cancer and led to poor prognosis [47]. Silencing of *SORBS1* promoted the EMT process and attenuated chemical drug sensitivity, and it is a potential inhibitor of metastasis in cancer [57]. We inputted the tree DEGs in the miRDB, TargetScan and miWalk databases to identify the key miRNAs for the prognosis of endometriosis. As Figure 7 shows, *ITGA7* had 45 miRNAs, *ITGBL1* had 92 miRNAs, *SORBS1* had 159 miRNAs, hsa-miR-6745 was the only expressed miRNA between *ITGA7* and *ITGBL1*, and there were six expressed miRNAs between *ITGBL1* and *SORBS1*. We conjectured that overexpressed hsa-miR-6745 may be associated with poor outcomes and high recurrence of endometriosis. Although all three genes were upregulated, through literature data, we found that silencing of *SORBS1* may promote the progression of disease in most cancers; thus there may be some other regulatory relationship between *ITGBL1* and *SORBS1*. We cannot say that the six miRNAs may have a certain relationship with the prognosis of endometriosis.

In survival analysis, only *IGHM* had significance in OS and DFS. *IGHM* is a protein-coding gene. IgM antibodies are involved in the recognition and elimination of precancerous and cancerous lesions, have been found to be upregulated in breast cancer [58] and were considered a biomarker for recurrence [59]. *IGHM* also retained a significant prognostic impact on the density of intratumoural CD20+ B cells [60] and was associated with type 1 diabetes [61]. *IGHM* is involved in oxidative stress and in skin regeneration [62], suggesting that it may be involved in cell proliferation. We tried to find relevant IGHM-regulated cascade response signals, similar to other studies [63–67]. Transcription factor binding to *IGHM* enhancer 3 (TFE3) is related to renal cell carcinoma [68,69]. PRCC-TFE3 tRCC is a TFE3 Xp11.2 translocation renal cell carcinoma (TFE3-tRCC) that promotes cell survival and proliferation by implicating in PINK1-PRKN/parkin-dependent mitophagy and activating the expression of the E3 ubiquitin ligase PRKN, leading to rapid PINK1-PRKN-dependent mitophagy that promotes cell survival under mitochondrial oxidative damage as well as cell proliferation by decreasing mitochondrial ROS formation [68], suggesting that there are similar regulatory mechanisms in endometriosis. In our study, IGHM was significantly involved in the CC of extracellular exosomes. Exosomes are released following the fusion of multivesicular bodies with the plasma membrane and the extracellular release of intraluminal vesicles [70]. Exosomes are EVs 50–100 nm in size that deliver proteins, lipids and nucleic acids [71,72] to target cells, and their main functions include antigen presentation, pathogen spread, proliferation, differentiation, apoptosis, migration and invasion [73–75]. In our immunofluorescence validation, the expression of *IGHM* was highest in ectopic endometrium, and differed from eutopic endometrium and normal endometrium (Figure 5), which is consistent with our analysis. Therefore, regulating *IGHM* may be another method for endometriosis. We could not find any miRNAs that had been confirmed to interact with *IGHM* in the three miRNA databases, possibly indicating that *IGHM* may be a new biomarker for us to explore in the future.

## Conclusions

Above all, *ITGA7*, *ITGBL1* and *SORBS1* may be associated with cell proliferation, invasion and migration of endometriosis, hsa-miR-6745 may be a potential miRNA biomarker, and its high expression may be associated with poor prognosis. *IGHM* might be a potential target gene for the recurrence of endometriosis; however, to date, there have been no studies on *IGHM* in the reproductive system. Further research is needed to elucidate the role of this new target gene in endometriosis, and *ITGA7*, *ITGBL1*, *SORBS1* and *IGHM* may be therapeutic target genes. All drugs need to be validated by molecular biology or animal studies in future research.

## Data Availability

The datasets that support the findings of this study are openly available in GEO database at http://www.ncbi.nlm.nih.gov/geo/.
